# Supplementary figures and images for: Bio-Functionalized Ultra-Thin, Large-Area and Waterproof Silicone Membranes for Biomechanical Cellular Loading and Compliance Experiments
Source: Polymers (Basel). 2022 May 30;14(11):2213. doi: 10.3390/polym14112213 (PMC9182891; doi:10.3390/polym14112213)

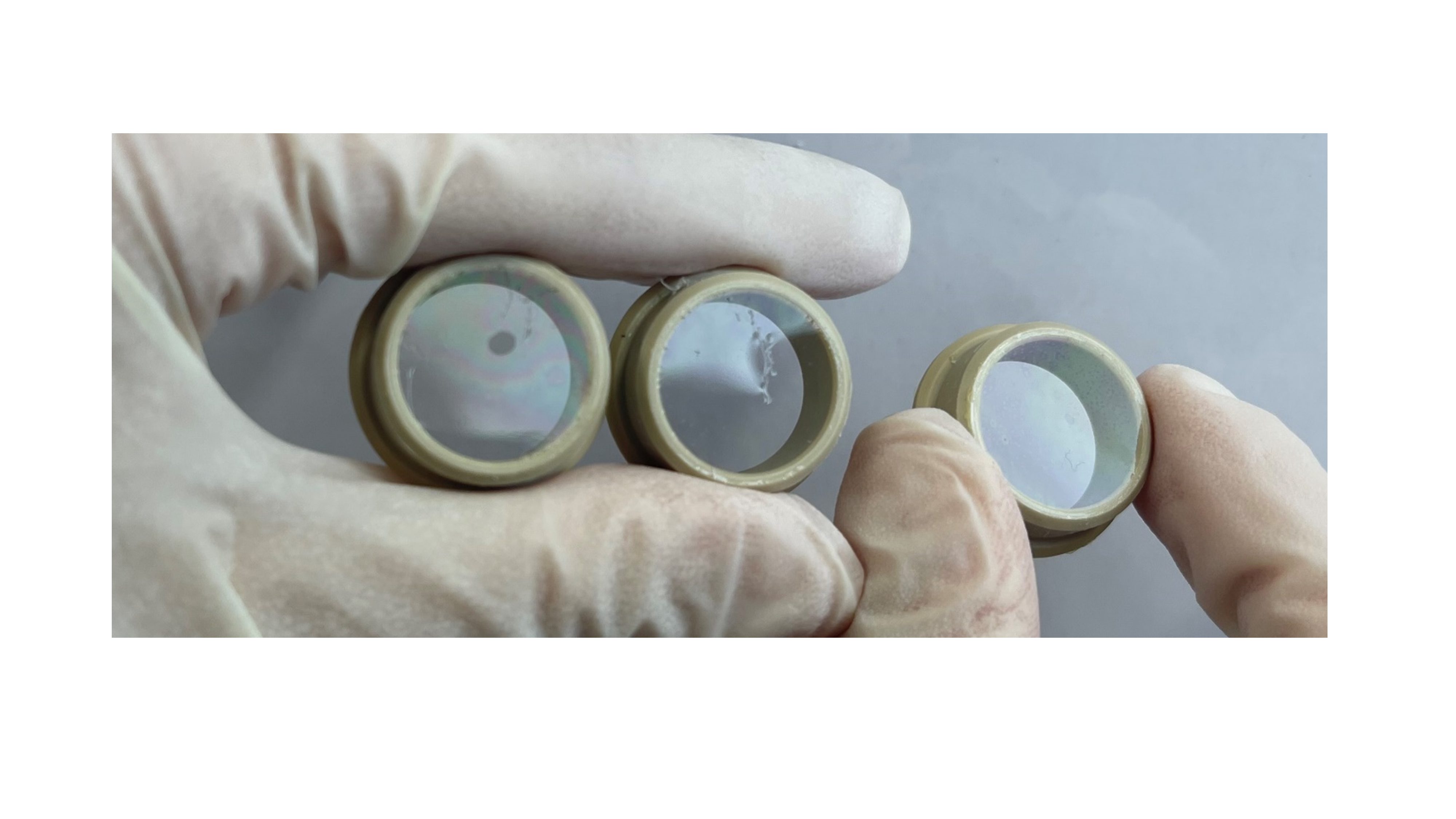

Supplement: Supplementary file 1 [file polymers-14-02213-s001.zip › Supplemantary Figure S1.tif]

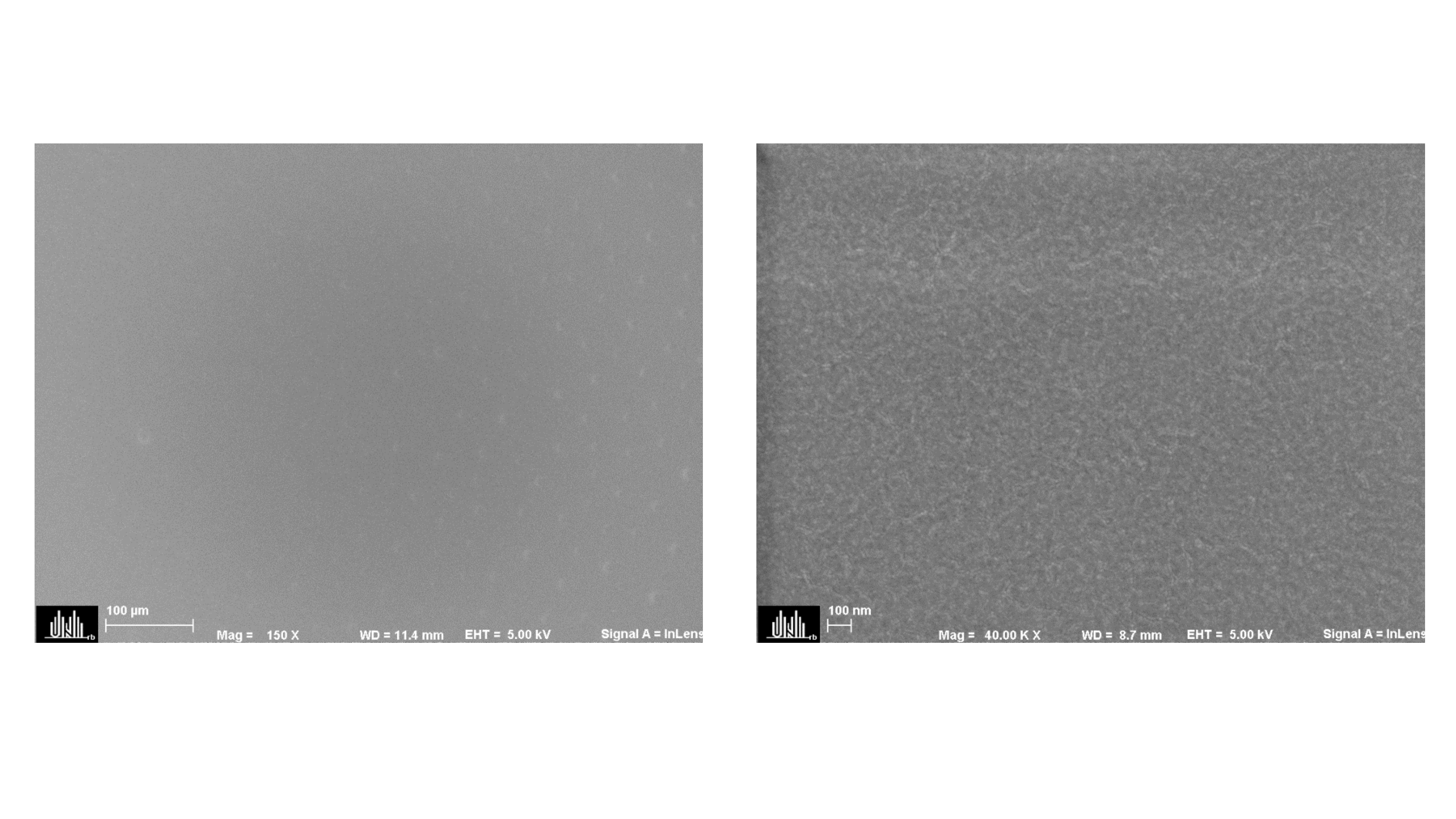

Supplement: Supplementary file 1 [file polymers-14-02213-s001.zip › Supplemantary Figure S3.tif]

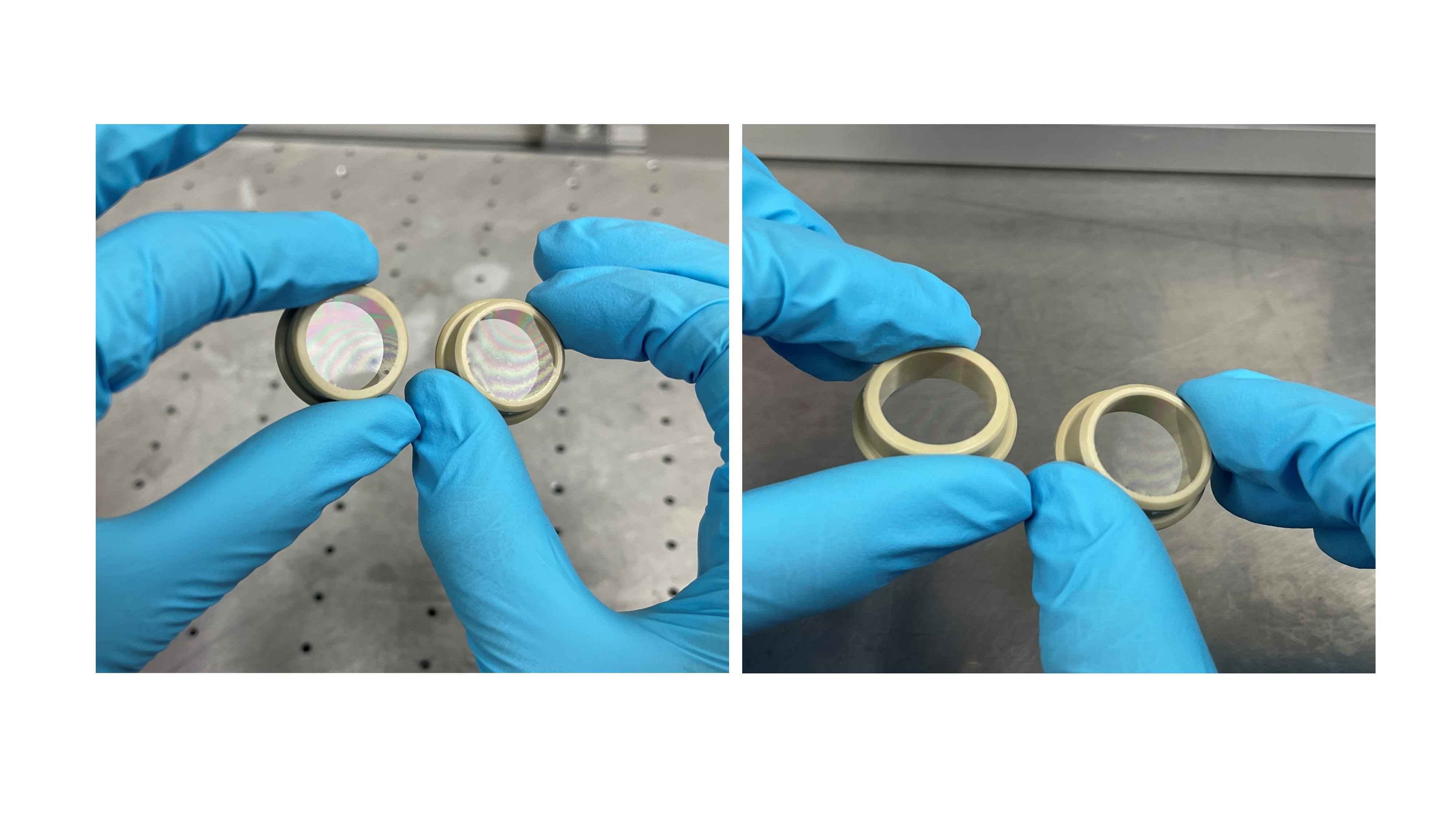

Supplement: Supplementary file 1 [file polymers-14-02213-s001.zip › Supplementary Figure S2.tif]
